# Influence of Milling Time on the Homogeneity and Magnetism of a Fe_70_Zr_30_ Partially Amorphous Alloy: Distribution of Curie Temperatures

**DOI:** 10.3390/ma13020490

**Published:** 2020-01-20

**Authors:** Alejandro F. Manchón-Gordón, Jhon J. Ipus, Javier S. Blázquez, Clara F. Conde, Alejandro Conde

**Affiliations:** Dpto. Física de la Materia Condensada, ICMSE-CSIC, Universidad de Sevilla, P.O. Box 1065, 41080 Sevilla, Spain; afmanchon@us.es (A.F.M.-G.); jhonipus@us.es (J.J.I.); cfconde@us.es (C.F.C.); conde@us.es (A.C.)

**Keywords:** mechanical alloying and milling, amorphous and nanocrystalline materials, soft magnetic materials, Mössbauer spectroscopy

## Abstract

In this work, the mechanically alloyed Fe_70_Zr_30_ (at. %) composition has been used to study the influence of milling time on its homogeneity and magnetic properties. The microstructure and Fe environment results show the formation of an almost fully amorphous alloy after 50 h of milling in a mixture of pure 70 at. % Fe and 30 at. % Zr. The soft magnetic behavior of the samples enhances with the increase of the milling time, which is ascribed to the averaging out of the magnetocrystalline anisotropy as the crystal size decreases and the amorphous fraction increases. The formation of a non-perfectly homogenous system leads to a certain compositional heterogeneity, motivating the existence of a distribution of Curie temperatures. The parameters of the distribution (the average Curie temperature, $\bar{T_{C}}$, and the broadening of the distribution, $∆T_{C}$) have been obtained using a recently reported procedure, based on the analysis of the approach towards the saturation curves and the magnetocaloric effect. The decrease of $∆T_{C}$ and the increase of $\bar{T_{C}}$ with the milling time are in agreement with the microstructural results. As the remaining α-Fe phase decreases, the amorphous matrix is enriched in Fe atoms, enhancing its magnetic response.

## 1. Introduction

The physical behavior of amorphous materials can differ from their crystalline counterparts, because of the nonexistence of long-range order translation symmetry. However, they present a short-range order (<10 Å) and, in some cases, medium-range order (10–30 Å) [1]. New phenomenology arises when the characteristic microstructural length becomes similar to the characteristic lengths of different phenomena (e.g., exchange length in the magnetic properties [2]). Consequently, amorphous alloys have been extensively studied in the last few decades because of their interesting physical properties (magnetic, mechanical, etc.) from a fundamental and technological point of view [3].

In particular, amorphous Fe–Zr alloys show singular magnetic phenomena at a low temperature [4,5], and present extremely different magnetic properties depending on the Fe content [6,7] and the local atomic order [8]. Although amorphous alloys have been generally produced by rapid quenching techniques [3], the presence of two eutectic points in the Fe–Zr binary phase diagram [9,10] limits rapid solidification procedures from producing completely amorphous alloys near the compositions of Fe_90_Zr_10_ and Fe_25_Zr_75_. Therefore, other ways to produce amorphous samples out of the eutectic compositions have been used, such as mechanical alloying (MA) [11], vapor-deposition techniques [12], or sputtering techniques [13,14]. For example, amorphous samples can be produced for an Fe content in the range of 20–93 at. %. and 30–78 at. % using sputtering techniques [13] and MA [15], respectively.

MA has become a very versatile technique to directly produce metastable microstructures (nanocrystalline, amorphous, etc.) [16] from compositional heterogeneous (generally pure elements) powder. During the milling process, the powders are continuously exposed to different phenomena, such as fracture and cold welding or/and intensive plastic deformation, which refine the powder morphology and define the microstructure of the final products. Therefore, the starting material becomes homogenized after milling, forming an alloy, which is generally a strongly disordered system. In this sense, the milling time has a crucial role in the final properties of the products. For example, an increase in the milling time could cause the ordering and crystallization of the amorphous matrix [17,18], leading to a complete change of the properties of the system. Residual stress [19], the existence of contamination in the milling media [20], or the formation of a non-perfectly homogeneous system are other important factors affected by the milling time [21].

A wide variety of amorphous alloy compositions have been prepared by MA [22,23]. However, several aspects inherent to powder samples affect the magnetic properties of the samples produced by this technique. The existence of two different length scales, the powder particle size and the nanometric scale of the crystalline structures, combine to describe the soft magnetic behavior in mechanically alloyed powders [24]. For example, the coercivity is one order of magnitude higher than those of similar compositions obtained by rapid quenching, because of the increase of the magnetoelastic anisotropy [25,26]. On the other hand, in the case of the magnetocaloric effect, a general decrease in the peak value, ascribed to the Curie transition of the amorphous phase, and a large broadening of the thermal dependence of the magnetic entropy change are observed, because of the presence of inhomogeneities in the amorphous matrix [27,28]. These inhomogeneities lead to the existence of a non-negligible distribution of Curie temperatures [29]. Therefore, in order to optimize the magnetic behavior of a material obtained by ball milling, it is necessary to study the influence of the milling time on the structure and magnetic properties of the powders.

^57^Fe Mössbauer spectroscopy has been frequently used to follow the compositional evolution of the Fe containing phases present in mechanically alloyed powders [21,30]. In fact, it is known that Mössbauer spectroscopy (MS) is a more sensitive technique than X-ray diffraction for the detection of small fractions of Fe phases when paramagnetic and ferromagnetic phases coexist [15]. Moreover, MS can provide extensive and valuable information, such as the oxidation state, the coordination mode, and the nature of ligands [31], as well as the composition of the nanocrystals [30]. Finally, the determination of the magnetic ordering temperature allows for determining the coexistence or the absence of different phases in the studied system [32].

Recently, a procedure has been reported to obtain the average Curie temperature and the broadening of the distribution from the analysis of the approach to the saturation curves in almost fully amorphous alloys [33]. In this work, this procedure has been applied to partially amorphous Fe_70_Zr_30_ alloys, and their microstructural and magnetic properties have been studied as a function of the temperature and milling time.

## 2. Materials and Methods

An amorphous alloy with an Fe_70_Zr_30_ at. % composition was prepared by high-energy ball milling, starting from high purity elemental powders of iron and zirconium (>99.9%). The milling process was carried out for a maximum of 50 h at 350 rpm in a planetary ball mill Fritsch Pulverisette Vario 4 (Fritsch GmbH, Idar-Oberstein, Germany). Further milling parameters can be found elsewhere [21].

The microstructure was studied at room temperature by X-ray diffraction (XRD) using Cu–Kα (λ_Kα1_ = 1.5406 Å) radiation in a Bruker D8 I diffractometer (Bruker, Karlsruhe, Germany). A scanning electron microscopy (SEM) Jeol 6460 LV (JEOL Ltd., Tokyo, Japan), operated at 30 kV and working in secondary electrons mode, was used to get a better understanding of the morphology of the samples. The powder particle size was obtained by averaging over a minimum of 50 particles for each sample.

The magnetic properties were measured as a function of the temperature, using a maximum applied field of $\mu_{0}H_{app}$ = 1.5 T in a Lakeshore 7407 (Lakeshore, Westerville, OH, USA) vibrating sample magnetometer. The saturation magnetization ($M_{S}$) and paramagnetic susceptibility ($\chi_{p}$) were obtained by fitting the experimental high-field magnetization curve to the law of approach to saturation [34]. The magnetic entropy change ($∆S_{M}$) was calculated from isothermal magnetization curves, using the Maxwell relation, as follows:

(1) $$∆S_{M}=\mu_{0}\overset{H}{\underset{0}{\int }}{\left(\frac{\partial M}{\partial T}\right)}_{H}dH$$

where T is the temperature and H is the maximum magnetic field. All of these magnitudes have been obtained after correcting the demagnetizing factor using a density of 7205 kg/m^3^; details of the procedure can be found elsewhere [33].

The local environment of Fe atoms was analyzed as a function of the temperature by Mössbauer spectrometry (MS) in transmission geometry using a ^57^Co(Rh) source (WissEl, Starnberg, Germany). The values of the hyperfine parameters were obtained by fitting the measured spectra with the NORMOS program (Normos-90, WissEl, Starnberg, Germany) [35].

## 3. Results and Discussion

### 3.1. Microstructural Characterization

**Figure 1:**
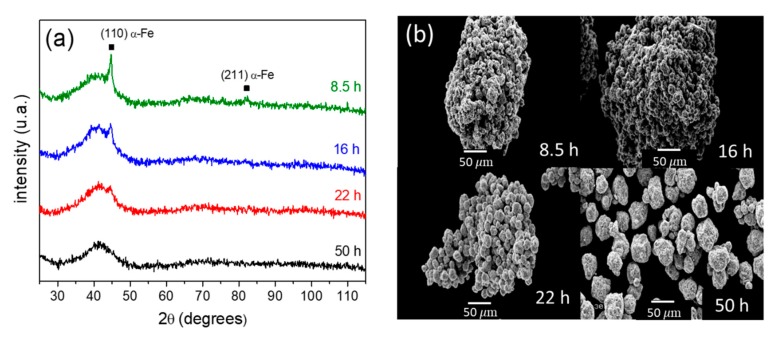
(**a**) XRD patterns and (**b**) scanning electron microscopy images in secondary electron modes after selected milling times.

The X-ray diffraction patterns of the samples after some selected milling times are shown in Figure 1a. The milling process started from pure crystalline α-Fe and hcp-Zr phases. However, the peaks attributed to the hcp-Zr phase were not detected for these milling times, showing that this phase became imperceptible as the Zr atoms dissolved in the Fe amorphous matrix. This fact is evidenced by the appearance of a wide amorphous halo near 2θ = 42°, and reflects the absence of a long-range order in the studied samples. On the other hand, the α-Fe(Zr) (110) maximum was only clearly observed after shorter milling times of 8.5 and 16 h. The intensity of this crystalline peak decreased as the milling time increased, and after 50 h of milling, no traces of the crystalline phase were detected.

The microstructural modifications induced by the milling process can be observed from a direct comparison of the SEM micrographs working in the secondary electrons mode (Figure 1b). The increase of the milling time induced the disintegration of the agglomerates observed at lower milling times, which led to the isolation of the powder particles. After milling for 50 h, the powder particles reached 23 ± 11 µm. The uncertainty represents the standard deviation.

**Figure 2:**
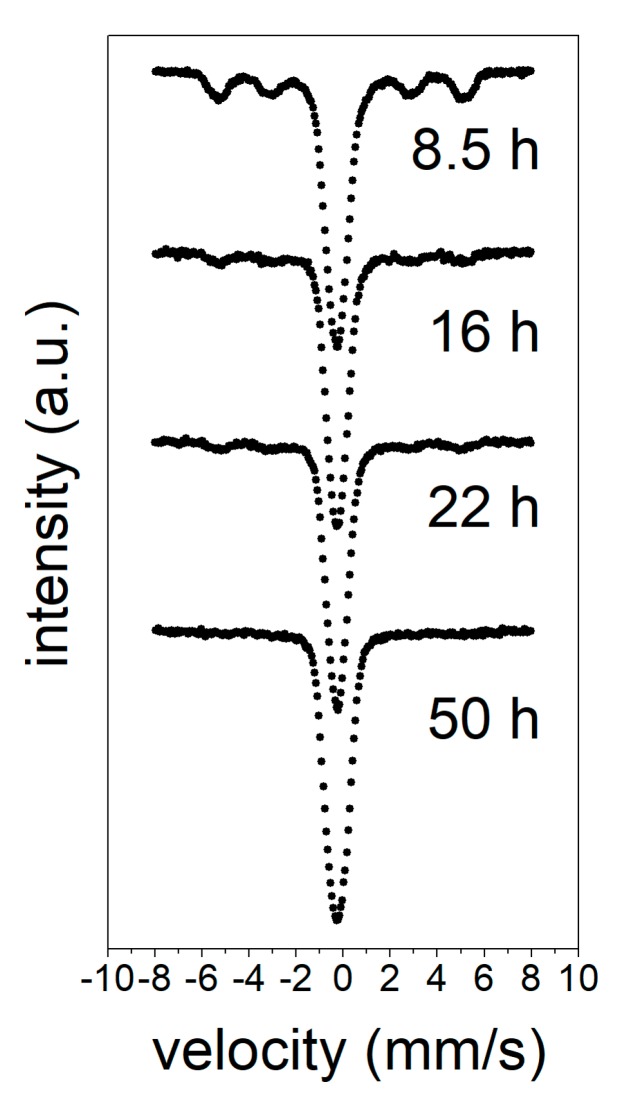
Room temperature Mössbauer spectra collected on the samples after different milling times.

**Figure 3:**
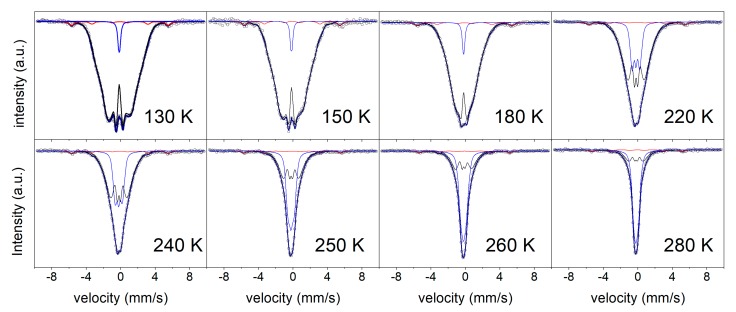
Mössbauer spectra of the sample after 50 h of milling, collected at different temperatures. The symbols correspond to the experimental data. Blue, black, and red subspectra correspond to Fe atoms in the paramagnetic, ferromagnetic, and α-Fe phases, respectively.

Figure 2 shows Mössbauer spectra performed at room temperature after the selected milling times. The decrease of the intensities of the ferromagnetic ordered phase, associated with the α-Fe phase, as the milling time increased, shows the progress of the amorphization process. As the milling time increased, a decrease of the peaks of around ±4 and ±6 mm/s, and the appearance of a wide quadrupole could be followed, indicating the formation of a paramagnetic phase at room temperature. After 50 h of milling, the spectrum consisted only of a broad quadrupole distribution, and no traces of any magnetically-ordered phase were detected. This is in agreement with the XRD results, showing a completely amorphous sample. However, because of the broad doublets and high line widths observed after this time of milling, the Mössbauer spectra as a function of the temperature were measured in order to distinguish the coexistence of different local environments or distinct phases (Figure 3).

Three components have been used to fit the spectra as a function of temperature, namely: a distribution of hyperfine fields to describe the broad sextets assigned to the ferromagnetic amorphous sample, a distribution of quadrupole splitting to describe the paramagnetic contribution, and one crystalline ferromagnetic site to describe the residual α-Fe(Zr) phase (not detected at room temperature). In order to avoid the strong overlapping between the low hyperfine magnetic field and quadrupolar distributions at T < 200 K, a unique doublet was successfully used to fit the paramagnetic contribution. Whether there is a unique sharp magnetic ordering temperature cannot be distinguished, but their distribution is a more realistic description of the system. Although the XRD patterns and MS at room temperature did not show the presence of α-Fe crystallites in the sample after 50 h of milling, it is evidenced in the MS spectra at lower temperatures (~3% phase contribution, see Figure 3). The ferromagnetic character of the amorphous matrix at low temperatures allows for a ferromagnetic coupling between the residual α-Fe nanocrystals (responsible for the visible contribution in the MS). At room temperature, this contribution disappeared in MS, as the amorphous matrix became paramagnetic and prevented the coupling of the disseminated α-Fe nanocrystals, which exhibited a superparamagnetic behavior.

**Figure 4:**
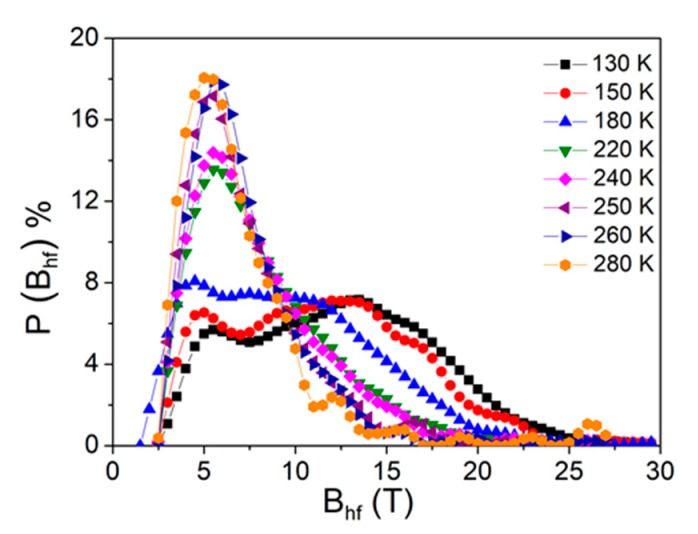
Hyperfine field distributions for the Mössbauer spectra collected at the indicated temperatures.

The hyperfine field distribution, $P\left(B_{hf}\right)$, is shown in Figure 4. Two humps can be observed for the low temperatures, in which the low field peak originated from Fe-deficient sites, the richest in Zr atoms, and the high field hump corresponds to Fe-rich sites. The high field contributions decrease as the temperature increases, while the low field contributions clearly increase. This behavior of $P\left(B_{hf}\right)$ is consistent with the existence of different local Fe environments in the amorphous matrix, as well as a progressive transition from ferromagnetic to paramagnetic behavior. The hyperfine parameters obtained from the fit of the Mössbauer spectrum at the studied temperatures are summarized in Table 1. The decrease of the mean value of the hyperfine field as the temperature increased is clear, evidencing the broad ferro–paramagnetic transition of the sample.

### 3.2. Magnetic Characterization

**Figure 5:**
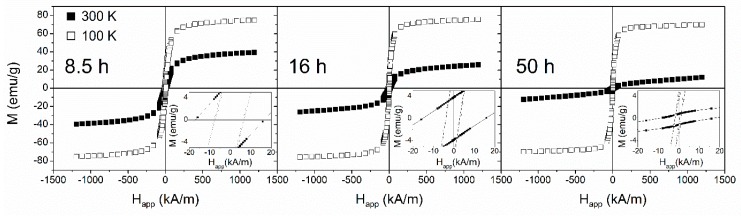
Magnetic hysteresis loops, taken at 100 (open symbols) and 300 K (solid symbols), of the mechanically alloyed Fe_70_Zr_30_ powders for the selected milling times. The inset shows the low field region of the hysteresis loops.

**Figure 6:**
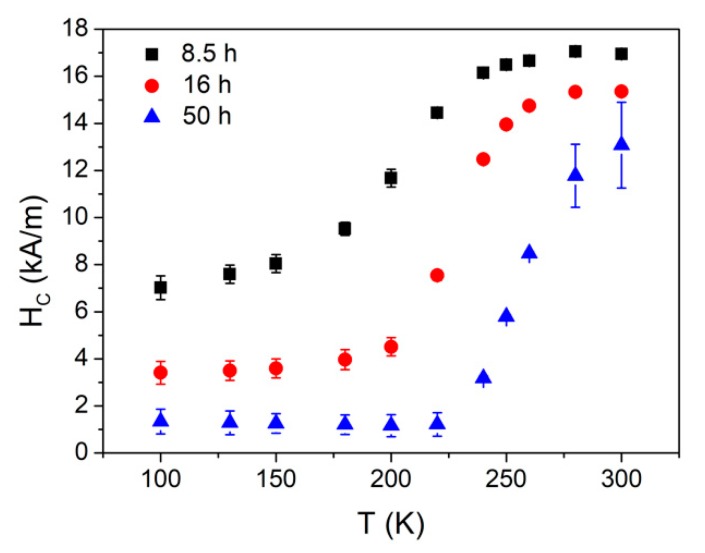
Coercivity as a function of temperature after 8.5, 16, and 50 h of milling.

Figure 5 shows the magnetic hysteresis loops $M\left(H_{app}\right)$, where $H_{app}$ is the applied field, recorded at 100 and 300 K for some selected milling times; the insets show the expanded region close to zero field. The loops exhibited similar behavior at 100 K, characterized by low coercive fields, $H_{C}$, (<10 kA/m) which decreased as the milling time increased. Figure 6 shows the coercivity as a function of the temperature for the same selected milling times. $H_{C}$ continuously increased with the temperature for the sample milled for 8.5 h, but it remained almost constant at a low temperature for the samples milled for 16 and 50 h. For the latter samples, a clear $H_{C}$ increase was observed at approximately 220 K because of the ferromagnetic transition of the amorphous phase. The lowest $H_{C}$ value was obtained at low temperatures, and corresponded to 50 h of milling—the almost fully amorphous sample. The averaging out of the magnetocrystalline anisotropy as the crystal size decreased, and the ferromagnetic character of the amorphous matrix as the milling time increased can explain the enhancement of the soft magnetic behavior of the samples. However, the magnetoelastic anisotropy ascribed to a long-range scale (in the range of the powder particle size) prevented a decrease in coercivity below 1 kA/m [26].

The saturation magnetization, $M_{S}$, and paramagnetic susceptibility, $\chi_{p}$, were obtained by fitting the experimental high-field magnetization curve to the law of approach to saturation [34], as follows:

(2) $$M=M_{S}\left(1-\frac{a}{H}-\frac{b}{H^{2}}\right)+\chi_{p}H,$$

where H is the internal field and a and b are the constant coefficients. Theoretically, the value of parameter a is a measure of the structural inhomogeneities caused by defects within the magnetic substances [36,37], and b is related to the effective anisotropy [38].

**Figure 7:**
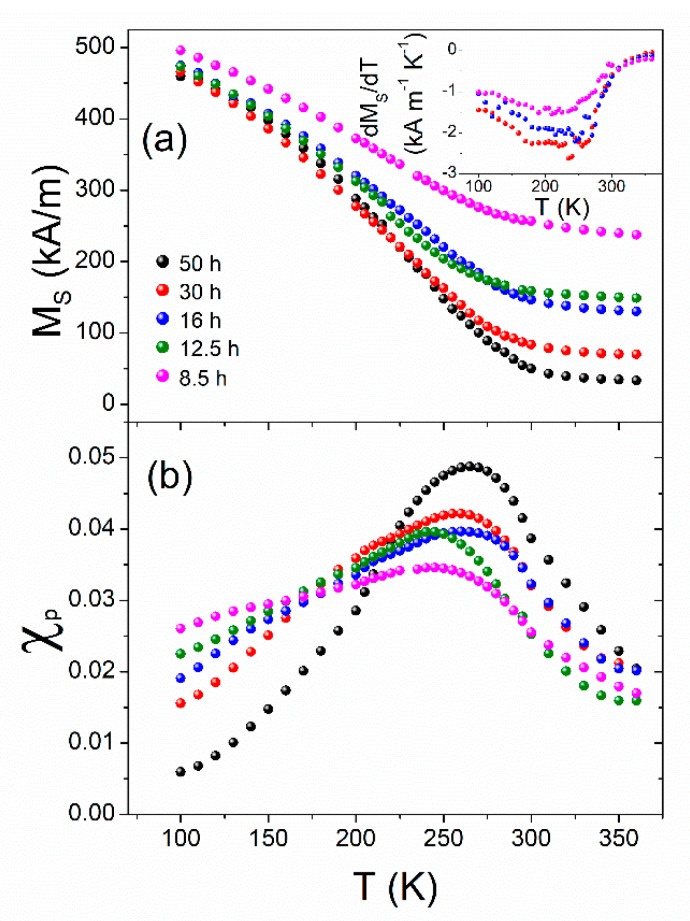
(**a**) Saturation magnetization and (**b**) paramagnetic susceptibility curves from the law of approach to saturation after different times of milling.

A nonlinear least square fitting procedure was carried out to fit the experimental magnetization at a high field ($M/M(\mu_{0}H_{app}=1.5\;T)>$ 0.8), considering a, $M_{S}$, and $\chi_{p}$ as free fitting parameters, using Equation (2). The extracted values of $M_{S}$ are displayed in Figure 7a as a function of the temperature after different times of milling. $M_{S}$ continuously decreased with the increasing temperature, because of the progressive change from the ferromagnetic to paramagnetic character of the amorphous phase. The decrease of $M_{S}$ at a high temperature as the milling time increased is in agreement with the gradual amorphization of the sample. The Curie temperature ($T_{C}$) is usually approximated to the inflection point, at low fields, $T_{inf}$, of the magnetization, as a function of temperature curves. The inset of Figure 7a shows that $T_{inf}$ is not well-defined, suggesting the presence of inhomogeneities in the samples, in agreement with the above results.

The temperature dependence of the paramagnetic susceptibility, $\chi_{p}$, is shown in Figure 7b. As the milling time increased, the $\chi_{p}$ curves became narrower, and a shift of the peak temperature ($T_{pk}^{\chi }$) towards higher temperatures, along with an increase of the peak intensity, can be observed.

**Figure 8:**
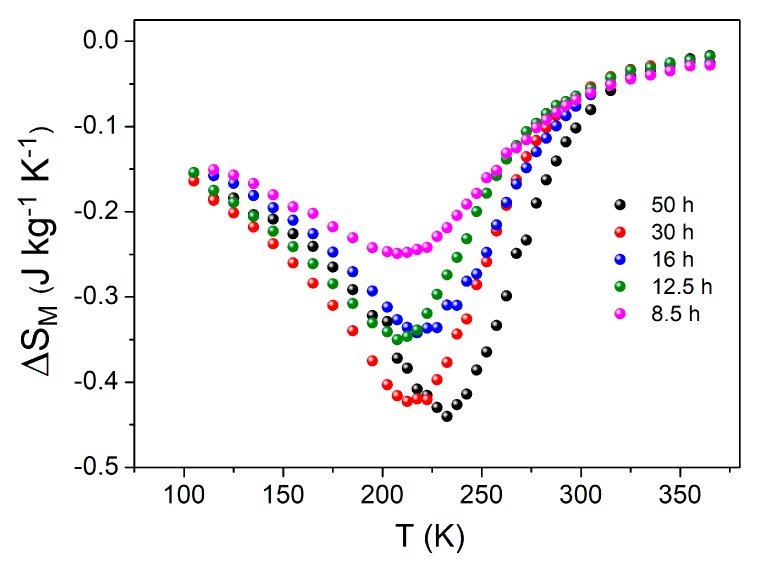
Magnetic entropy changes at $\mu_{0}∆H$ = 1 T after correcting the demagnetizing field for samples after different times of milling.

Figure 8 shows the magnetic entropy change after the selected milling times for a maximum field change of 1 T, as a function of the temperature. As the milling time increased, an increase of $\left|∆S_{M}\right|$ can be observed, because of the increase of the amorphous fraction, reaching a maximum value of about 0.45 J·kg^−1^K^−1^ after 50 h of milling.

All $H_{C}$, $M_{S}$, $\chi_{p}$, and $∆S_{M}$ curves show a continuous transition, instead of the abrupt transition expected for a theoretically pure system. Indeed, the typical compositional inhomogeneities of the samples produced by ball milling could lead to a distribution of their physical properties. The knowledge of the parameters characterizing those distributions helps to obtain a more realistic description of the behavior of the samples. In the case of the Curie temperature distribution, the average Curie temperature, $\bar{T_{C}}$, and the broadening of the distribution $∆T_{C}$ can be obtained by the following:

(3) $$T_{inf}-\bar{T_{C}}=-0.732(6)∆T_{C}$$

(4) $$T_{pk}^{\chi }-\bar{T_{C}}=0.502(23)∆T_{C}-0.0039(7)∆T_{C}{}^{2}$$

(5) $$T_{pk}^{MCE}-\bar{T_{C}}=-0.709∆T_{C}+0.0014\;∆T_{C}{}^{2},$$

where $T_{inf}$ and $T_{pk}^{\chi }$ have been already defined, and $T_{pk}^{MCE}$ is the temperature corresponding to the maximum magnetic entropy change for $\mu_{0}∆H_{app}$ = 1 T.

$\bar{T_{C}}$ and $∆T_{C}$ can be obtained by combining two of the three above equations. However, as previously reported, $T_{inf}$ is poorly defined. For this reason, Equations (4) and (5), along with the experimental values of $T_{pk}^{\chi }$ and $T_{pk}^{MCE}$, were selected to determine the parameters of the distribution of the Curie temperatures (see Table 2). A continuous increase of $\bar{T_{C}}$ can be observed as the milling time increased. This behavior is in agreement with the progressive Fe enrichment of the amorphous matrix as the amorphous fraction increased and the α-Fe(Zr) crystalline phase fraction decreased. It is known that the Curie temperature is strongly dependent on the Fe content in the FeZr system in this compositional range. In fact, $T_{C}$ increased as the Fe concentration increased until 85 at. % Fe [13]. $∆T_{C}$ remained constant, at ~40 K, and only after 50 h of milling (almost fully amorphous sample), did it decrease to 35 K. Therefore, the width of the distribution seemed to be almost constant when the amorphous and crystalline phases clearly coexisted.

## 4. Conclusions

The magnetic and microstructure properties of a partially amorphous Fe_70_Zr_30_ alloy as a function of the milling time and temperature have been studied. The formation of the amorphous structure has been verified using X-ray diffraction and Mössbauer spectrometry results. After 50 h milling, the presence of a residual α-Fe phase is only detected by Mössbauer spectrometry at temperatures below the Curie temperature of the amorphous phase. At temperatures above the Curie temperature of the amorphous phase, tiny α-Fe phase nanocrystals become superparamagnetic, and thus the ferromagnetic contribution disappears.

The increase of the milling time enhances the soft magnetic behavior of the samples. The compositional heterogeneity of the samples, typical of those produced by mechanical milling, leads to a distribution of Curie temperatures, for which the parameters have been determined. The average Curie temperature continuously increases as the amorphous phase becomes enriched in Fe. The broadening only decreases after 50 h of milling, when the sample is almost fully amorphous.

## Figures and Tables

**Table 1:** Hyperfine parameters of the as-milled amorphous alloy after 50 h of milling. Area% is the relative contribution of the component, and $\bar{B_{hf}}$ and $\bar{\delta }$ are the mean values of the hyperfine magnetic field and the isomer shift of the distribution of hyperfine fields, respectively.

Temperature (K)	Component	Area%	$\bar{B_{hf}}\;\pm \;0.1\;(T)$	$\bar{\delta }\;\pm \;0.02\;(\mathrm{mm/s})$
130	Hyperfine distribution	94.0	12.4	−0.17
	α-Fe site	3.0	-	-
150	Hyperfine distribution	94.0	11.5	−0.18
	α-Fe site	2.9		
180	Hyperfine distribution	92.6	9.7	−0.20
	α-Fe site	3.3		-
220	Hyperfine distribution	77.5	8.3	−0.22
	α-Fe site	0.1	-	-
240	Hyperfine distribution	69.5	8.0	−0.22
	α-Fe site	0.1	-	-
250	Hyperfine distribution	47.7	7.3	−0.23
	α-Fe site	0.1	-	-
260	Hyperfine distribution	37.5	7.3	−0.23
	α-Fe site	0.1	-	-
280	Hyperfine distribution	26.5	7.1	−0.23
	α-Fe site	0.1	-	-

**Table 2:** Experimental values of the temperatures corresponding to the peaks of paramagnetic susceptibility, $T_{pk}^{\chi }$, and the maximum magnetocaloric effect, $T_{pk}^{MCE}$, along with the average value of the Curie temperature, $\bar{T_{C}}$, and the width of the distribution, $∆T_{C}$, obtained by Equations (4) and (5) for different times of milling.

Milling Time (h)	$T_{pk}^{\chi }$ (±5 K)	$T_{pk}^{MCE}$ (±2.5 K)	$\bar{T_{C}}$ (±4 K)	$∆T_{C}$ (±5 K)
8.5	245	207.5	234	40
12.5	250	212.5	239	40
16	255	217.5	244	40
30	260	222.5	249	40
50	265	237.5	261	35
